# Combination of Micro-Corrugation Process and Pre-Stretched Method for Highly Stretchable Vertical Wavy Structured Metal Interconnects

**DOI:** 10.3390/mi13081210

**Published:** 2022-07-29

**Authors:** Michitaka Yamamoto, Shinji Okuda, Seiichi Takamatsu, Toshihiro Itoh

**Affiliations:** 1 Department of Precision Engineering, Graduate School of Engineering, The University of Tokyo, 7-3-1 Hongo, Bunkyo-ku, Tokyo 113-8656, Japan; yamamoto-michitaka@g.ecc.u-tokyo.ac.jp (M.Y.); takamatsu@pe.t.u-tokyo.ac.jp (S.T.); 2 Department of Human and Engineered Environmental Studies, Graduate School of Frontier Sciences, The University of Tokyo, Kashiwa 277-8563, Japan; sin0ku.skn@gmail.com

**Keywords:** stretchable interconnects, Cu foil, micro-corrugation process, pre-stretch method

## Abstract

Metal interconnects with a vertical wavy structure have been studied to realize high-density and low-electric-resistance stretchable interconnects. This study proposed a new method for fabricating vertical wavy structured metal interconnects that comprises the pre-stretch method and the micro-corrugation process. The pre-stretch method is a conventional method in which a metal film is placed on a pre-stretched substrate, and a vertical wavy structure is formed using the return force of the substrate. The micro-corrugation process is a recent method in which a metal foil is bent vertically and continuously using micro-gears. In the proposed method, the pitch of the vertical wavy structured interconnect fabricated using the micro-corrugation process is significantly narrowed using the restoring force of the pre-stretched substrate, with stretchability improvement of up to 165%, which is significantly higher than that of conventional vertical wavy structured metal interconnects. The electrical resistance of the fabricated interconnect was low (120–160 mΩ) and stable (±2 mΩ or less) until breakage by strain. In addition, the fabricated interconnect exhibits durability of more than 6500 times in a 30% strain cycle test.

## 1. Introduction

With the recent advancement of the Internet of things devices and sensors, there is a high demand for devices attached to humans, and stretchable devices that can expand and contract in response to human movement are required. For example, flexible displays [1], skin sensors [2,3], and smart gloves [4,5,6,7] have been developed. Connecting electrical components based on stretchable interconnects is one of the common ways to fabricate stretchable devices because interconnects account for a large part of these devices. In particular, stretchable interconnects with repeated stretchability of more than 30% are required because the strain of the skin due to human motion is up to approximately 30%, except in some areas where it varies greatly.

Several types of methods for fabricating stretchable interconnects have been reported, such as stretchable conductive inks [8,9,10], nanostructured metal particles [11,12], liquid metals [13], buckled structures [14,15], and two- or three-dimensional spring-like structured metals [16,17,18,19,20,21,22,23,24,25,26,27,28]. The spring-like structured metal type has merit in electrical stability. The wavy structure is one of the typical two- or three-dimensional spring-like structures, and when processing metals in a wavy shape, two types of structures are possible: a horizontal wavy structure in which a wave is formed in the in-plane direction of the substrate [16,17,18,19,20,21,22], and a vertical wavy structure in which a wave is formed in the vertical direction of the substrate [23,24,25,26,27,28,29]. A horizontal wavy structure is easy to process and exhibits a high stretchability of 100% or more [19,22]. However, to achieve high stretchability, a wide interconnect of nearly 1 mm width is required, which increases the area occupied by the interconnect. On the other hand, the vertical wavy structure can produce high-density interconnections. However, the pre-stretch method, where a metal film is placed on a pre-stretched substrate and a vertical wavy structure is formed using the return force of the substrate [23,24], is the standard method for fabricating vertical wavy structures, making it difficult to stably produce them. In addition, although the stretchability of metal interconnects with a vertical wavy structure created using the pre-stretch method was approximately 100% at the highest [24], the thin metal film was made thinner to improve stretchability, which improved the electrical resistance (from several tens to several hundreds of ohms).

In recent years, the micro-corrugation process, where micro-gears continuously bend metal foils, has been proposed [29]. In the micro-corrugation process, the wave shape can be controlled by the gear shape and the distance between the gears. Thus, stable vertical wavy structure fabrication was achieved. On the other hand, the stretchability of interconnects fabricated using the micro-corrugation process is approximately 60%, because the size of the gear is large and the aspect ratio of the height and pitch of the wave cannot be increased. To improve stretchability, the wave structure needs to be folded more, meaning that the aspect ratio of the height and pitch of the wave should be increased.

This paper proposes a new method in which a metal film is bent in advance through the micro-corrugation process and then attached to a pre-stretched substrate. In the proposed method, the pitch of a vertical wave fabricated through the micro-corrugation process is significantly narrowed by using the restoring force of the pre-stretched substrate, meaning that the aspect ratio between the height and pitch of the wave is increased. In this study, gears of different sizes were prepared to fabricate different wave shapes, and the improvement due to pre-stretching was evaluated by varying the pre-stretch rate. Furthermore, strain cycle tests were conducted to evaluate durability.

## 2. Experiment Method

### 2.1. Fabrication Method

**Figure 1:**
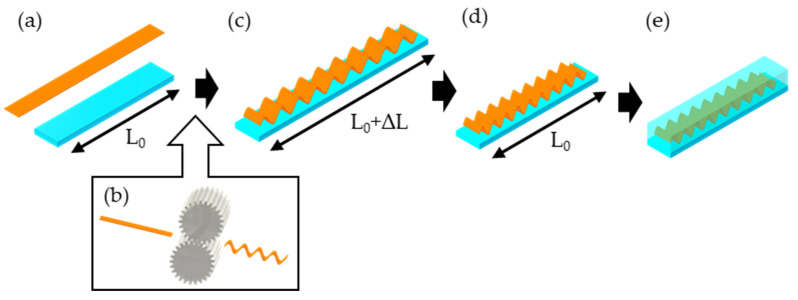
The proposed fabrication method of stretchable interconnects with vertical wavy structure, comprising the pre-stretch method and micro-corrugation process: (**a**) preparation of Cu foil and silicon rubber substrate; (**b**) a pair of gears continuously bending Cu foil; (**c**) micro-corrugated Cu foil is bonded and cured on the pre-stretched silicone rubber substrate; (**d**) the substrate is returned to its original length; and (**e**) the entire interconnect is embedded in silicone rubber.

Figure 1 shows the proposed fabrication method for stretchable interconnections with vertical wavy structures fabricated by using the pre-stretch and micro-corrugation methods. Cu foil and silicon rubber substrate were prepared, and a pair of gears continuously bent the Cu foil to form a vertical wavy structure. The micro-corrugated Cu foil was bonded to the pre-stretched silicone rubber substrate using silicone rubber as an adhesion layer, and then the substrate was returned to its original length. Finally, the entire interconnect was embedded in silicone rubber.

**Figure 2:**
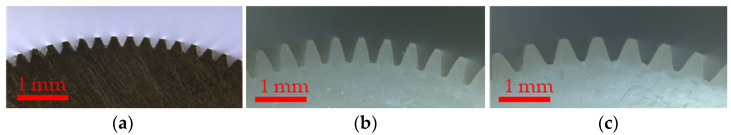
Types of gears used in the corrugating process: (**a**) module 0.1, (**b**) module 0.15, and (**c**) module 0.2.

In this study, three types of involute gears with modules 0.1, 0.15, and 0.2 were prepared as gears for the micro-corrugation process to create and investigate different wave shapes (aspect ratio, angle, height, etc.). The geometry of each gear is shown in Figure 2. Note that the material and the gear pressure angle of module 0.1 are different from those of modules 0.15 and 0.20 because of the difference in the machining method. The distance between gears was optimized for each module; however, other conditions were unified in this paper. Optimal conditions and the optimal module may change if the thickness or width of the Cu foil is changed.

The Cu foil with a film thickness of 5 µm was cut into 3 mm in width and 100 mm in length. KE-1316 (Shin-Etsu Chemical Co., Ltd., Tokyo, Japan) was the silicone rubber used. The silicone rubber was cured by heating it at 70 °C for 30 min. Vacuum defoaming was performed before curing. The initial thickness of the silicone rubber was 0.4 mm. The percentage of pre-stretch ratio compared to the initial length (Δ*L/L*_0_) was set to 0%, 25%, 50%, and 75%. The thickness of the sample after being embedded in the silicone rubber was approximately 1 mm. Note that when a Cu foil with a film thickness of 10 µm was used or when the pre-stretch ratio was set to more than 100%, the Cu foil peeled off from the base silicone rubber, and the sample could not be fabricated.

### 2.2. Evaluation Method

The wavy structure after sample preparation was observed by cross-sectional scanning electron microscopy (SEM, VHX-D510, Keyence Corp., Osaka, Japan). Stretchability was evaluated using a tensile measurement machine (FTN1-13A, Aikoh Engineering Co., Ltd., Aichi, Japan) equipped with a load cell (Model-3020, Aikoh Engineering Co., Ltd., Aichi, Japan) and a tensile test fixture. The tensile speed was set to 30 mm/min. A linear gauge (GS-5100A, Ono Sokki Co., Ltd., Kanagawa, Japan) and a gauge counter (DG-2310, Ono Sokki Co., Ltd., Kanagawa, Japan) were used to measure displacement during the tensile test. Resistance was also measured with the four-terminal method using a source meter (2400, Keithley Instruments Inc., Cleveland, OH, USA). Durability to repeated tensile strain was evaluated by repeating the reciprocating tensile force up to a predetermined strain using the above apparatus. The tensile and return speed was set to 300 mm/min.

## 3. Experiment Results

### 3.1. Fabricated Structure Observation and Prediction of Stretchability by Geometric Calculation

**Figure 3:**
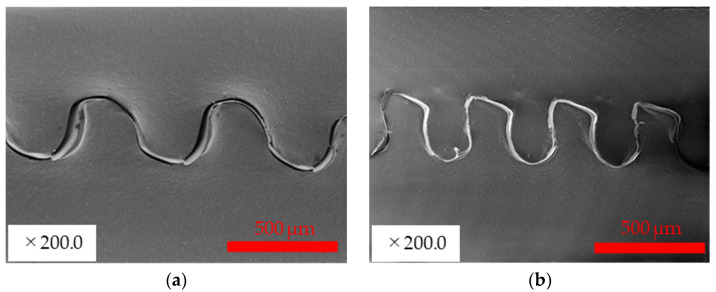
Example of SEM cross-sectional view of the created interconnect: (**a**) fabricated using gears with module 0.2 and 0% pre-stretch; (**b**) fabricated using gears with module 0.2 and 75% pre-stretch.

**Figure 4:**
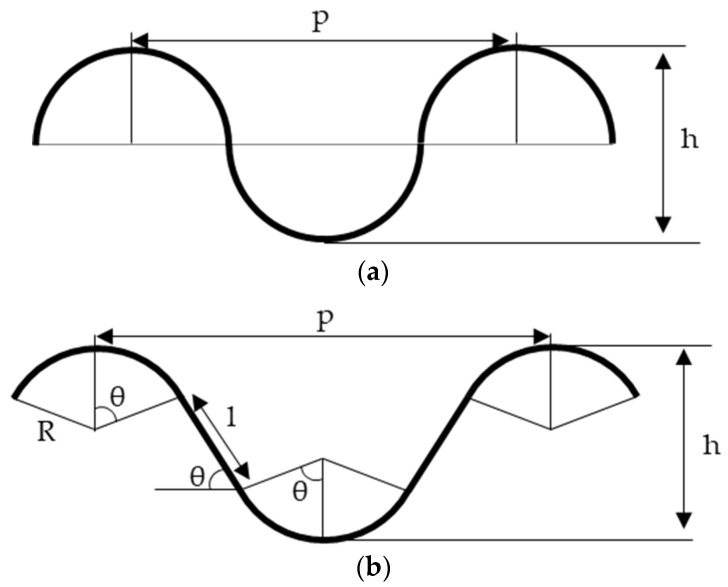
Geometric models for stretchability: (**a**) conventionally used sine-wave-based model; (**b**) a model close to the geometry of the proposed method.

Examples of SEM cross-sectional images of the fabricated interconnects are shown in Figure 3. Figure 3 shows that the proposed method succeeded in fabricating a continuous wavy structure. Note that the minor structural damage observed in these SEM images seems to be caused by the cutting process for preparing the SEM observation. Figure 3 also shows that by incorporating the pre-stretch method into the proposed method, the wave height remains the same, and only the wave pitch is narrowed compared to the sample fabricated without the pre-stretch method. Stretchability can be predicted on the basis of the shape of the wave by calculating the length of the wave based on geometric calculations. In previous studies, the following equation predicted stretchability, assuming that the wave is a sine wave, as shown in Figure 4a [17,24,29].

(1) $$S=\frac{n\times \int_{0}^{p}\sqrt{1+{\left(\frac{\pi h}{p}\cos\frac{\pi x}{p}\right)}^{2}}dx}{L}-1,$$

where *S*, *p*, and *h* represent the stretchability, pitch, and height of waves, respectively, and *L* represents the length of the structure.

On the other hand, Figure 3b shows that the wavy shape fabricated using the proposed method is not a clean sine wave but a model, as shown in Figure 4b, consisting of a series of circular arcs and a straight portion. Considering the geometric conditions for the wave pitch *p* and height *h*, the following equation holds for the model in Figure 4b:

(2) p=4Rsinθ+2lcosθ,

(3) h=2R(1−cosθ)+lsinθ,

where *R* represents the radius of the arc, *l* represents the length of the linear portion, and *θ* represents the angle between the linear portion and arc. The length *L_1_* for one cycle in this model is

(4) $$L_{1}=4R\theta +2l,$$

and the limit of stretchability *S* is calculated using the one cycle length *L*_1_ and the pitch *p* of the wave as the following equation:

(5) $$S=\frac{L_{1}-p}{p},$$

By transforming Equation (5) using Equations (2)–(4), the following equation can be obtained:

(6) $$S=\frac{\theta \sin\theta }{1-\cos\theta }+\frac{2\sin\theta -2\theta \cos\theta }{1-\cos\theta }a-2,$$

where a represents the aspect ratio (=*h*/*p*). Note that the angle *θ* and aspect ratio *a* need to satisfy the following equation:

(7) $$\tan^{-1}\left(2a\right)<\theta <2\tan^{-1}\left(2a\right).$$

**Figure 5:**
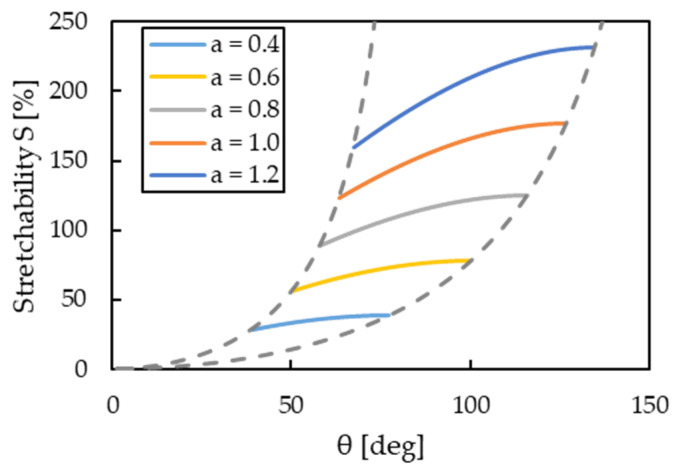
Graphical illustration of Equation (6).

A graphical representation of Equation (6) is shown in Figure 5. Although only the effect of the aspect ratio *a* has been mentioned in the previous study, Figure 5 shows that angle *θ* also contributes to stretchability in addition to aspect ratio *a*, especially in the case of a large aspect ratio.

**Figure 6:**
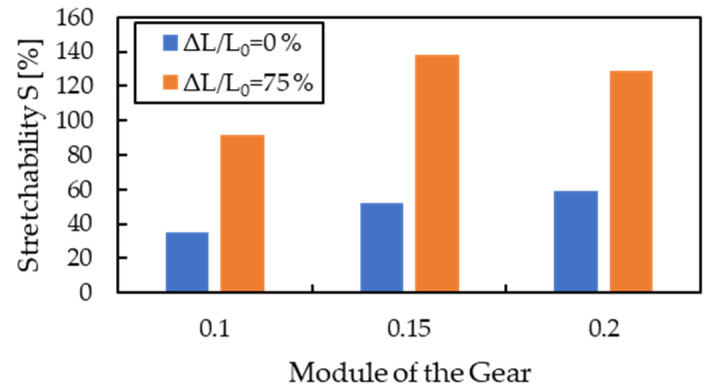
Prediction of stretchability based on SEM observed value in changing the gear module and with or without pre-stretch.

Table 1 summarizes the parameters of wavy structures extracted from the SEM cross-sectional images when using gears with a pitch of 0.1, 0.15, and 0.2 and pre-stretch ratios of 0% and 75%, respectively. The prediction of the stretchability of each wavy structure calculated by Equation (6) is shown in Figure 6. Figure 6 shows that the proposed method is expected to improve stretchability by two times or more because of increased aspect ratio *a* and angle *θ*.

### 3.2. Evaluation of Stretchability

**Figure 7:**
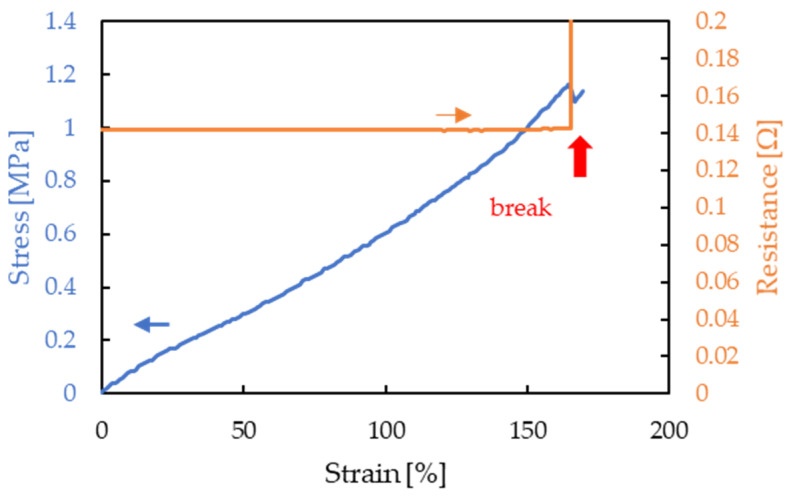
Stretchability evaluation of interconnect fabricated using the proposed method.

Figure 7 shows an example of the stretchability evaluation of an interconnect fabricated using the proposed method. In all samples, resistance was low, ranging from 120 to 160 mΩ, and the resistance was stable with a change of less than ±2 mΩ until breakage by strain. The stress at breakage was approximately 1.2 MPa. The pressure was calculated by dividing the stress applied to the interconnect by the cross-sectional area of the entire interconnect, representing the total cross-sectional area of the Cu foil and silicone rubber.

**Figure 8:**
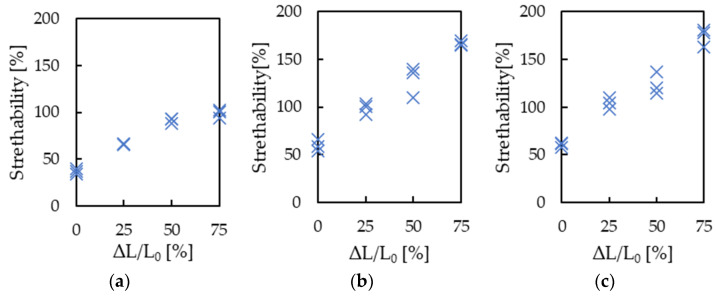
Results of evaluation of stretchability S for each sample made with different types of gear and pre-stretch ratios: (**a**) gears with module 0.1, (**b**) gears with module 0.15, and (**c**) gears with module 0.2.

The results of stretchability S for each sample prepared with different gear types and pre-stretch ratios are shown in Figure 8. Figure 8 indicates that stretchability improves as the pre-stretch ratio increases for any gear type. When gears with module 0.15 or 0.2 were used and the pre-stretch ratio was set to 75%, 165% stretchability was realized. The stretchability of 165% is larger than the value predicted from the wave’s shape. This could be attributed to the silicone rubber. Peeling between silicone rubber and metal interconnects effectively improves stretchability [22]. Furthermore, thin Cu foil undergoes plastic deformation of more than several percent under certain conditions [30], which may have improved the stretchability of the interconnects.

### 3.3. Cycle Test

**Figure 9:**
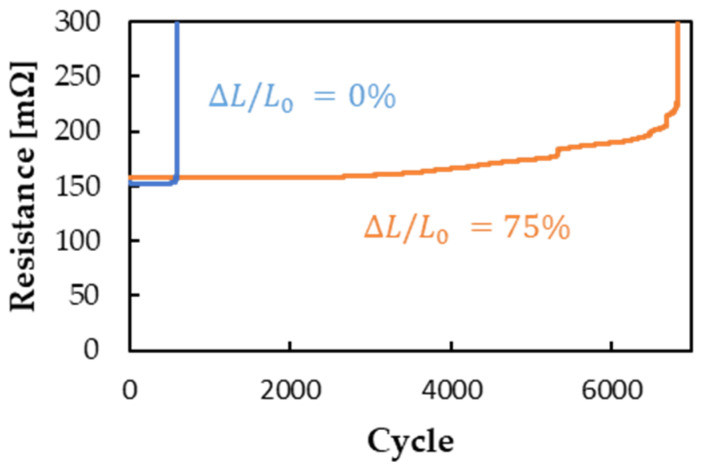
The result of the cycle test with strain up to 30% for samples prepared using gears with module 0.15 and a pre-stretch ratio of 0% or 75%.

The cycle test results with strain up to 30% for samples prepared using gears with module 0.15 and a pre-stretch ratio of 0% or 75% are presented in Figure 9. The sample with a 0% pre-stretched ratio failed after approximately 550 cycles, whereas the sample with a 75% pre-stretched ratio exhibited a significant improvement, withstanding more than 6500 cycles. This improvement seems to be achieved because of the improvement in stretchability.

## 4. Discussion

This paper proposes a new method in which a metal film is bent in advance through the micro-corrugation process and then attached to a pre-stretched substrate. The proposed method has merit because after creating a stable vertical wavy structure using the micro-corrugation process, the wave’s pitch is expected to be significantly improved by the restoring force of the pre-stretched substrate. Table 2 compares the proposed method with previous studies on wavy structured metal interconnects. Table 2 shows that the interconnect fabricated using the proposed method has the lowest resistance and the best stretchability among other metal interconnects with a wavy structure.

On the other hand, although the proposed method significantly improved stretchability, the sample fabricated using it still failed after approximately 6500 cycles in the 30% strain cycle test. Fabricating interconnects with higher stretchability and creating a structure that prevents the concentration of stress are considered as effective approaches to increase the number of cycles that samples can withstand during stretchability tests. For example, there was a report in which a sample withstood more cycles than our sample by covering the Cu interconnects by polyimide, even though the report’s maximum stretchability was lower than ours. In the previous study, Cu interconnects with a 100 μm width were covered by polyimide with a 200 µm width, and cycle tensile tests were conducted at a rate of 10%/s [31]. Protecting the top and bottom of the Cu foil with another material also seems to be an effective method for increasing the number of cycles that the interconnect fabricated using the proposed method can withstand.

## 5. Conclusions

In this study, we proposed a new method for fabricating vertical wavy structures that comprises the pre-stretch method and the micro-corrugation process. The interconnect fabricated using the proposed method exhibited a maximum of 165% stretchability, significantly higher than that of the conventional vertical-wavy-structured metal interconnect. The electrical resistance of the fabricated interconnect was low, ranging from 120 to 160 mΩ, and the resistance was stable with a change of less than ±2 mΩ until breakage by strain. Furthermore, the fabricated interconnect withstood more than 6500 cycles in a 30% strain cycle test. The proposed method is expected to be useful for wiring stretchable devices.

## Figures and Tables

**Table 1:** The measured value of each wavy structure fabricated by micro-corrugation process and with or without pre-stretch.

Gear Module	Pre-Stretch Ratio (Δ*L/L*)	Pitch (μm)	Height (μm)	Aspect Ratio	*θ* (°)
0.10	0%	300	120	0.40	58
	75%	190	130	0.68	86
0.15	0%	470	240	0.49	70
	75%	270	240	0.89	95
0.20	0%	600	310	0.52	70
	75%	370	310	0.84	129

**Table 2:** Comparison between the proposed method and previous studies on wavy structured metal interconnects.

Structure	Fabrication Method	Material (Thickness)	Wave Shape		Resistance (Length)	Stretch Ability	Authors
			Pitch	Height			
Horizontal wavy structure	Photolithography	Au (100 nm)	80 μm	40 μm	-	54%	Gray, D.S. et al. (2004) [17]
		Au (4 μm)	2 mm	1 mm	5.58 Ω (30 mm)	72%	Brosteaux, D. et al. (2007) [16]
		Au (4 μm)	500 μm	700 μm	2 Ω/cm	100%	Gonzalez, M. et al. (2008) [19]
		Cu (2 03BCm)	2.6 mm	2.25 mm	-	135%	Hsu, Y.-Y. et al. (2010) [22]
	Laser patterning	Al (50 μm)	1.2–4.8 mm	1.2–3.6 mm	183 mΩ (30 mm)	70%	Marchiori, B. et al. (2018) [18]
Vertical wavy structure	Pre-stretch method	Au (25 nm)	8.4 μm	1.2 μm	7.5 Ω (4.6 mm)	28%	Lacour, S.P. et al. (2004) [23]
		Au (20 nm)	8.4 μm	1.2 μm	316 mΩ (25 mm)	100%	Jones, J. et al. (2004) [24]
	Metal deposition on wavy structured substrate	Ag (400 nm)	400 μm	200 μm	44 Ω (20 mm)	50%	Jeong, J. et al. (2009) [26]
	Micro-corrugation	Cu (5 μm)	300–600 μm	300 μm	-	60%	Yamamoto, M. et al. (2020) [29]
	Micro-corrugation and pre-stretch	Cu (5 μm)			120–160 mΩ (100 mm)	165%	This work

## References

[1] Sekitani T., Nakajima H., Maeda H., Fukushima T., Aida T., Hata K., Someya T. (2009). Stretchable Active-Matrix Organic Light-Emitting Diode Display Using Printable Elastic Conductors. Nat. Mater..

[2] Misery L., Loser K., Ständer S. (2016). Sensitive Skin. J. Eur. Acad. Derm. Venereol..

[3] Liu Y., Pharr M., Salvatore G.A. (2017). Lab-on-Skin: A Review of Flexible and Stretchable Electronics for Wearable Health Monitoring. ACS Nano.

[4] Lee J., Kim S., Lee J., Yang D., Park B.C., Ryu S., Park I. (2014). A Stretchable Strain Sensor Based on a Metal Nanoparticle Thin Film for Human Motion Detection. Nanoscale.

[5] Yan C., Wang J., Kang W., Cui M., Wang X., Foo C.Y., Chee K.J., Lee P.S. (2014). Highly Stretchable Piezoresistive Graphene-Nanocellulose Nanopaper for Strain Sensors. Adv. Mater..

[6] Aw K., Budd J., Wilshaw-Sparkes T. (2022). Data Glove Using Soft and Stretchable Piezoresistive Sensors. Micromachines.

[7] Takamatsu S., Minami K., Itoh T. (2021). Fabrication of Highly Stretchable Strain Sensor Fiber by Laser Slitting of Conductive-Polymer-Coated Polyurethane Film for Human Hand Monitoring. Sens. Mater..

[8] Larmagnac A., Eggenberger S., Janossy H., Vörös J. (2014). Stretchable Electronics Based on Ag-PDMS Composites. Sci. Rep..

[9] Merilampi S., Björninen T., Haukka V., Ruuskanen P., Ukkonen L., Sydänheimo L. (2010). Analysis of Electrically Conductive Silver Ink on Stretchable Substrates under Tensile Load. Microelectron. Reliab..

[10] Zhang Z., Zhang Y., Jiang X., Bukhari H., Zhang Z., Han W., Xie E. (2019). Simple and Efficient Pressure Sensor Based on PDMS Wrapped CNT Arrays. Carbon.

[11] Rosset S., Niklaus M., Dubois P., Shea H.R. (2009). Metal Ion Implantation for the Fabrication of Stretchable Electrodes on Elastomers. Adv. Funct. Mater..

[12] Corbelli G., Ghisleri C., Marelli M., Milani P., Ravagnan L. (2011). Highly Deformable Nanostructured Elastomeric Electrodes With Improving Conductivity Upon Cyclical Stretching. Adv. Mater..

[13] Jackson N., Buckley J., Clarke C., Stam F. (2018). Manufacturing Methods of Stretchable Liquid Metal-Based Antenna. Microsyst. Technol..

[14] Hu X., Dou Y., Li J., Liu Z. (2019). Buckled Structures: Fabrication and Applications in Wearable Electronics. Small.

[15] Lacour S.P., Chan D., Wagner S., Li T., Suo Z. (2006). Mechanisms of Reversible Stretchability of Thin Metal Films on Elastomeric Substrates. Appl. Phys. Lett..

[16] Brosteaux D., Axisa F., Gonzalez M., Vanfleteren J. (2007). Design and Fabrication of Elastic Interconnections for Stretchable Electronic Circuits. IEEE Electron Dev. Lett..

[17] Gray D.S., Tien J., Chen C.S. (2004). High-Conductivity Elastomeric Electronics. Adv. Mater..

[18] Marchiori B., Delattre R., Hannah S., Blayac S., Ramuz M. (2018). Laser-Patterned Metallic Interconnections for All Stretchable Organic Electrochemical Transistors. Sci. Rep..

[19] Gonzalez M., Axisa F., Bulcke M.V., Brosteaux D., Vandevelde B., Vanfleteren J. (2008). Design of Metal Interconnects for Stretchable Electronic Circuits. Microelectron. Reliab..

[20] Leng K., Guo C., Wu K., Wu Z., Leng K., Guo C., Wu K., Wu Z. (2018). Tunnel Encapsulation Technology for Durability Improvement in Stretchable Electronics Fabrication. Micromachines.

[21] Vanfleteren J., Gonzalez M., Bossuyt F., Hsu Y.-Y., Vervust T., De Wolf I., Jablonski M. (2012). Printed Circuit Board Technology Inspired Stretchable Circuits. MRS Bull..

[22] Hsu Y.-Y., Gonzalez M., Bossuyt F., Axisa F., Vanfleteren J., de Wolf I. (2010). The Effect of Pitch on Deformation Behavior and the Stretching-Induced Failure of a Polymer-Encapsulated Stretchable Circuit. J. Micromech. Microeng..

[23] Lacour S.P., Jones J., Suo Z., Wagner S. (2004). Design and Performance of Thin Metal Film Interconnects for Skin-Like Electronic Circuits. IEEE Electron Dev. Lett..

[24] Jones J., Lacour S.P., Wagner S., Suo Z. (2004). Stretchable Wavy Metal Interconnects. J. Vac. Sci. Technol. A.

[25] Wagner S., Lacour S.P., Jones J., Hsu P.I., Sturm J.C., Li T., Suo Z. (2004). Electronic Skin: Architecture and Components. Phys. E Low Dimens. Syst. Nanostruct..

[26] Jeong J., Kim S., Cho J., Hong Y. (2009). Stable Stretchable Silver Electrode Directly Deposited on Wavy Elastomeric Substrate. IEEE Electron Dev. Lett..

[27] Liu Y., Wang X., Xu Y., Xue Z., Zhang Y., Ning X., Cheng X., Xue Y., Lu D., Zhang Q. (2019). Harnessing the Interface Mechanics of Hard Films and Soft Substrates for 3D Assembly by Controlled Buckling. Proc. Natl. Acad. Sci. USA.

[28] Park C.W., Jung S.W., Lim S.C., Oh J.-Y., Na B.S., Lee S.S., Chu H.Y., Koo J.B. (2014). Fabrication of Well-Controlled Wavy Metal Interconnect Structures on Stress-Free Elastomeric Substrates. Microelectron. Eng..

[29] Yamamoto M., Karasawa R., Okuda S., Takamatsu S., Itoh T. (2020). Long Wavy Copper Stretchable Interconnects Fabricated by Continuous Microcorrugation Process for Wearable Applications. Eng. Rep..

[30] Simons G., Weippert C., Dual J., Villain J. (2006). Size Effects in Tensile Testing of Thin Cold Rolled and Annealed Cu Foils. Mater. Sci. Eng. A.

[31] Verplancke R., Bossuyt F., Cuypers D., Vanfleteren J. (2012). Thin-Film Stretchable Electronics Technology Based on Meandering Interconnections: Fabrication and Mechanical Performance. J. Micromech. Microeng..

